# Synchronous Leydig Cell Tumor and Seminoma in the Ipsilateral Testis

**DOI:** 10.1155/2018/8747131

**Published:** 2018-02-19

**Authors:** Ifeyinwa E. Obiorah, Alexandra Kyrillos, Metin Ozdemirli

**Affiliations:** Department of Pathology, MedStar Georgetown University Hospital, Washington, DC, USA

## Abstract

Leydig cell tumor is a rare sex cord tumor that accounts for 1–3% of all testicular neoplasms. Seminomas are more common and occur in 30–40% of testicular tumors. Leydig cell tumors are derived from undifferentiated gonadal mesenchyme and the concurrent development of the tumor and a seminoma which are derived from germinal epithelium in an ipsilateral testis is extremely rare. Here we report a case of ipsilateral Leydig cell tumor and seminoma occurring in a 38-year-old man with a left testicular mass. The key to diagnosis is dependent on histopathology and immunohistochemistry. To our knowledge, this is the first diagnosis of the two disease entities in a unilateral testis using immunohistochemistry. Increased awareness of the entity is important in order to distinguish Leydig cell tumor and seminomas from other malignancies due to difference in therapeutic management.

## 1. Introduction

Leydig cell tumor is an uncommon testicular tumor derived from the gonadal stroma. It occurs in all age groups, mostly in the third to sixth decades [1]. Leydig cell tumors may produce endocrine changes and can lead to feminizing or virilizing syndromes due to increased production of androgen and/or estrogens. Majority of these tumors follow a benign clinical course; however, 10% of the tumors are malignant [2]. Leydig cell tumors can be pure or mixed and can occur concurrently with other sex cord-stromal tumors or very rarely with germ cell tumors. The simultaneous occurrence of seminoma and Leydig cell tumor in the unilateral testis is extremely rare. To the best of our knowledge, there are only four cases reported in the literature [3–6]. The diagnosis of these cases was made on histological sections without the utilization of any immunohistochemistry. Sex cord-stromal tumors and clear cell carcinoma can show solid growth patterns with diffuse clear cell morphology which resemble seminoma [7] and differentiating between the disorders can be challenging. Although classic histological morphology can aid diagnosis, immunohistochemistry remains the key to definitive diagnosis.

## 2. Case Report

**Figure 1:**
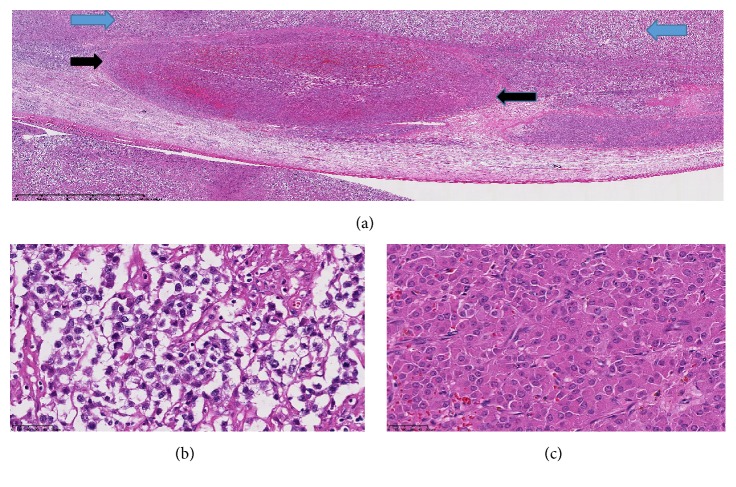
Histological examination of the left-sided testicular mass. Two distinct masses are identified. (a) The classic seminoma with clear cell morphology (depicted by the blue arrows) is on the top and the circumscribed Leydig cell tumor is at the bottom (black arrows) (hematoxylin and eosin (H&E), ×250). On higher magnification, (b) the seminoma cells contain abundant clear cytoplasm and slightly hyperchromatic nuclei (H&E, ×4000). (c) The Leydig cell tumor is composed of polygonal cells with prominent nucleoli with eosinophilic, granular, and vacuolated cytoplasm (H&E, ×4000).

**Figure 2:**
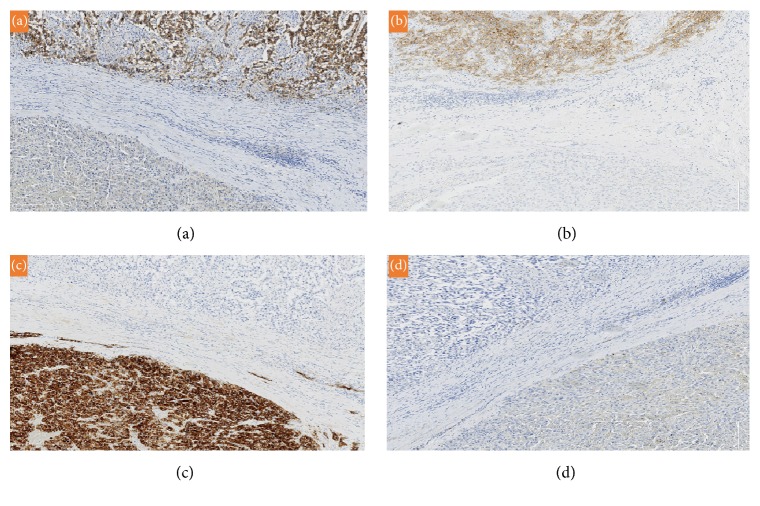
Immunohistochemical staining of the seminoma and Leydig cell tumor. (a) The seminoma cells (top) stained positively for (a) CD117 and (b) PLAP, while the Leydig cell tumor (bottom) was negative for both markers. (c) Inhibin immunostain is positive in the Leydig cell tumor (bottom) and negative in the seminoma (top). (d) Both the seminoma (top) and Leydig cell tumor (bottom) are negative for cytokeratin (×4000 each).

A 38-year-old male with no significant medical history presented at our institution with 5 months' history of increased left testicular swelling. Physical and ultrasound examination was suspicious for a testicular mass. Computed tomography scan of the abdomen was unremarkable and showed no lymphadenopathy. Preoperative hormone levels and tumor markers were unremarkable. A left radical inguinal orchiectomy was performed and the specimen was submitted for histopathological examination. Pathological examination revealed a well-circumscribed tan-pink fleshy mass with lobular appearance and focal hemorrhage measuring 6 cm and occupied 80% of the testis. A distinct second small tan-white nodule (1 cm) close to the tunica albuginea was also identified. Both masses were found alongside each other with intervening fibrous septa (Figure 1(a)). Histological sections of the first mass (Figure 1(b)) showed nests of tumor cells with clear cytoplasm with intervening fibrous bands and lymphocytes, which was consistent with a provisional diagnosis of seminoma. Microscopic examination of the small nodule (Figure 1(c)) revealed polygonal cells with eccentric nuclei, eosinophilic, granular, and vacuolated cytoplasm, mild atypia, and rare mitosis, which was consistent with a tentative diagnosis of a Leydig cell tumor. Based on the rarity of the provisional diagnosis, it was important to rule out other neoplasms such as a clear cell sex cord-stromal tumor or a clear cell carcinoma. On immunohistochemistry, neoplastic cells from the large mass were positive for CD117 (Figure 2(a)), placental alkaline phosphatase (PLAP) (Figure 2(b)), and CD10 and negative for inhibin (Figure 2(c)), cytokeratin (Figure 2(d)), *β*-catenin, smooth muscle actin (SMA), synaptophysin, desmin, S100, *β*-HCG, and *α*-fetoprotein. These results confirm the diagnosis of seminoma and exclude the diagnosis of a sex cord tumor or carcinoma. MIB-1 proliferative index was 80% in the seminoma cells. The Leydig cell tumor showed strong positivity for inhibin and vimentin and was negative for CD117, PLAP, cytokeratin, *β*-catenin, SMA, synaptophysin, desmin, S100, CD10, *β*-HCG, and *α*-fetoprotein. Approximately 10% of the tumor cells stained positively for MIB-1. Based on the findings, a diagnosis of a benign Leydig cell tumor was made. The immunohistochemical results supported the concurrent diagnosis of Leydig cell tumor and seminoma in a unilateral testis. The patient was followed up with imaging studies with no evidence of disease progression. The patient is currently stable, 10 years after surgery.

## 3. Discussion

Leydig cell tumors are rare testicular tumors that occur predominantly in the adult population. In children and adolescents, Leydig cell tumors are associated with precocious puberty and macrogenitosomia [8]. The adult patient is usually asymptomatic and typically presents with testicular enlargement. However, some patients may present with gynecomastia or decreased libido, which is usually related to the overproduction of estrogens. Majority of Leydig cell tumors are benign, but 10% of cases are malignant. No single pathologic criterion clearly defines a malignant Leydig cell tumor, but factors favoring a malignant behavior include large tumors (>5 cm), infiltrative borders, a high mitotic rate (>3 per high power field), cytologic atypia, vascular invasion, tumor necrosis [9], and extratesticular extension [10]. However, a tumor with predominantly benign features on gross or microscopic examination can metastasize and this occurs usually late in the course of the disease [8]. Seminomas are more common testicular tumors of germ cell origin. They either occur de novo or in association with other germ cell tumors such as yolk sac tumors or embryonal carcinoma. The coexistence of Leydig cell tumor, a gonadal stromal tumor, and seminoma, a germ cell tumor, in an ipsilateral testis is extremely rare. To date, only 5 cases (Table 1), including our patient, have been reported in the literature. A review of all the cases showed that the mean age of the patients was 33.8 ± 5.9 years. Only one case was associated with cryptorchidism and decreased libido due to elevated estrogen levels. The size of the Leydig cell tumors ranged from 1 to 1.5 cm, and their small sizes correlate with the benign nature of all 5 cases. All 5 cases were associated with seminoma; only one case had additional findings of embryonal carcinoma and choriocarcinoma. Three cases were treated with radical orchiectomy and 2 cases were treated with radical orchiectomy and adjuvant radiotherapy. For the 2 cases that reported outcomes, survival was 10 and 16 years, respectively.

Immunohistochemistry is a useful confirmatory tool that aids diagnosis of many diseases including cancer. Although morphologic histologic examination is the first step and can be used solely for diagnosis, unfortunately this can lead to misdiagnosis. Seminoma on histology without immunostaining can be confused with clear cell sex cord-stromal tumors which may also have a “water-clear” cytoplasm and a solid nested to diffuse arrangement of tumor cells which strongly resemble seminoma [2]. Inhibin is the most sensitive marker of Leydig cell tumors and is expressed in virtually all cases [11]. Leydig cell tumors are also positive for calretinin, Melan A, and vimentin and are negative for germ cell markers such as CD117, OCT3/4, PLAP, AFP, and *β*-HCG. MIB-1 proliferation index of ≥30% favors malignant potential [10]. On the other hand, seminoma stains positively for CD117, OCT3/4, CD10, and PLAP and negatively for inhibin, cytokeratin, AFP, and *β*-HCG [2, 10]. Although most cases of Leydig cell tumor are benign, some cases with low malignant potential have recurred with metastasis and it is important to distinguish them from seminoma because they do not generally respond to chemotherapy or radiotherapy. To our knowledge, this is the first case of synchronous Leydig cell tumor and seminoma using immunohistochemistry to confirm histological diagnosis.

The rare diagnosis of a synchronous Leydig cell tumor and seminoma can be made but it is important to distinguish these tumors from one another and from other malignancies due to different treatment strategies, especially in the event of recurrence. Confirmatory diagnosis with immunohistochemistry should be the diagnostic tool of choice, especially in challenging cases of testicular tumors.

## Figures and Tables

**Table 1:** Clinical summary of reported cases of synchronous Leydig cell tumor and seminoma in an ipsilateral testis.

Case	Age (years)	Associated clinical features	Mass size, seminoma/LCT	Associated GCN	Benign/malignant LCT	Treatment	Outcome Months (M)
1 [3]	34	None	3.2 cm/1.5 cm	Seminoma	Benign	Surgery, XRT	NA
2 [4]	39	Cryptorchidism and reduced libido	Total size, 1 cm	Seminoma	Benign	Surgery	NA
3 [5]	34	None	3.2 cm/1.2 cm	Seminoma	Benign	Surgery, XRT	16 years
4 [6]	24	None	3.5 cm/1 cm	Seminoma, EC, and CA	Benign	Surgery	NA
5 (present case)	38	None	6 cm/1 cm	Seminoma	Benign	Surgery	10 years

LCT, Leydig cell tumor; GCN, germ cell neoplasm; EC, embryonic carcinoma; CA, choriocarcinoma; XRT, radiotherapy; NA, not available.
